# Threshold Maps for Inclusion-Initiated Micro-Cracks and White Etching Areas in Bearing Steel: The Role of Impact Loading and Surface Sliding

**DOI:** 10.1007/s11249-018-1068-0

**Published:** 2018-07-30

**Authors:** T. Bruce, H. Long, R. S. Dwyer-Joyce

**Affiliations:** Department of Mechanical Engineering, The University of Sheffield, Sheffield, S1 3JD UK

**Keywords:** Rolling element bearing, Wind turbine gearbox, White etching cracking, White etching area, Rolling contact fatigue, Surface traction, Impact

## Abstract

Wind turbine gearbox (WTG) bearings can fail prematurely, significantly affecting wind turbine operational availability and the cost of energy production. The current most commonly accepted theory of failure mechanism is that the bearing subsurface is weakened by *white etching crack (WEC*) networks that eventually lead to the flaking away of material from the bearing surface. Subsurface damage due to rolling contact fatigue (RCF) is thought to be the main cause of premature failure, resulting from the initiation of micro-cracks, often at non-metallic inclusions or other material defects, which then propagate to the bearing surface. This study proposes a hypothesis that impact loading together with high levels of surface traction and contact pressure are important factors contributing to the initiation of micro-cracks and *white etching areas* (WEAs) at non-metallic inclusions which may lead to the formation of WEC networks. Both repeated impact and twin-disc RCF tests were designed to investigate inclusion-initiated micro-cracks and WEAs at subsurface in order to test this hypothesis. This led to the recreation of inclusion-initiated micro-cracks and WEAs in tested specimens, similar to the subsurface damage observed at inclusions in failed WTG bearing raceways. Tests were carried out to determine threshold levels of contact pressure, surface traction, and impact loading required for the formation of inclusion-initiated micro-cracks and WEAs.

## Introduction

Operational wind turbine (WT) availability is significantly affected by downtime caused by wind turbine gearbox (WTG) failures [1, 2]. Approximately two-thirds of these failures initiate in the bearings [3–6], despite the fact that they are designed to the same standards [7, 8] that satisfactorily predict bearing lifetime in many other industrial applications. They are also designed and manufactured according to recommended standards for WTG applications [9]. As average WT size increases, failure rates increase since larger turbine size leads to more flexible supporting structures that result in complex loading of turbine components [10, 11]. For larger bearings, crack initiation and propagation mechanisms can differ from those found in small bearings [5]. Additionally, larger bearings have a higher probability of material defects, such as non-metallic inclusions, being located in a critical near surface position, which increases the probability of damage initiation at that defect location.

Bearing raceways experience a fatigue cycle each time a roller element passes over a contact point on its surface, which results in repeated stress cycles under rolling contact fatigue (RCF) [12–14]. The process eventually leads to bearing failure caused by material loss from the surface, or spalling, flaking of material particles from the surface of bearing raceways or rollers. Cracks usually initiate below the raceway surface at depths close to the location of the maximum shear stress, before propagating upwards, but may initiate at the surface in applications where there are large tangential shear stresses [14, 15]. Two modes of premature failure have been observed in WTG bearings, namely, white structure flaking (WSF), also known as irregular white etching area (IrWEA); and axial cracking of bearing raceways [3, 4, 16–18]. Both failure modes are thought to be linked to the development of WEC networks in material just beneath the raceway contact which may be formed at the so-called butterfly cracks, an established damage feature found in rolling element bearings [17, 19, 20]. Failures due to WSF and axially propagating WEC networks have been found to lead to WTG bearing failure within 5–10% of their L_10_ design life [10, 19]. The mechanism by which WEC networks lead to WTG bearing failure is the subject of intensive research [4, 12, 21–28], but the subject is complex and there are still no prediction methods for calculating remaining useful bearing life in WTG applications [3, 6, 10, 16, 17].

The main difference between WTGs and the gearboxes used in many other industrial applications is that WTGs step up shaft rotation from low speed, high input torque to high speed, low output torque; whereas most other gearboxes operate in the reverse direction of transmission. The result is that WTGs operate against high referred inertia from the generator that is attached to the high-speed end of the gearbox. High transient loads from the rotor blades are therefore absorbed by the gearbox, and consequently the bearings are loaded in an extreme manner that is relatively unique to WTGs. Additionally, wind turbines operate in a corrosive environment with salt water ingress to the gearbox, and are subjected to harsh and fluctuating wind conditions leading to subsequent turbine operational controls, resulting in transient loading conditions occurring frequently which lead to short duration over-loading, under-loading, and impact loading on gearbox components. These transient loading conditions may cause high levels of stress, especially concentrated at material defects such as non-metallic inclusions. The resulting stress levels can exceed the raceway material yield strength causing local yielding in the bearing subsurface and initiating microstructural alterations that lead to the development of WECs [10, 24]. The high variability of wind conditions and subsequent turbine controls lead to connections and disconnections between the generator and grid, causing the gearbox to experience frequent torque reversals, overloads, and even operations at under-loading conditions [10, 29–31].

During torque reversal and transient loading conditions, the acceleration and deceleration of the WT drivetrain can cause impact events at WTG bearings. Such events are most serious when bearing rolling elements are in the unloaded zone, where they may be instantaneously loaded beyond the material yield strength in misaligned conditions, along one or two contact points in the load profile [10]. A recent investigation focused on measuring the operational conditions in the high-speed stage of a WTG has reported that, when tested at lower torque and speed than the rated conditions, the measured speeds of the bearing cage and rolling elements are 30 ~ 40% lower than their theoretical values, indicating significant sliding of rolling elements over the raceways, even under steady-state conditions [32]. These periods of heavy and dynamic loading lead to transient raceway stresses sometimes exceeding 3.1 GPa and generator engagements and disengagements can lead to stresses up to 2.5–4 times higher than those during normal operating conditions [5], well above the yield strength of bearing steels [6]. At such high contact stresses, failure may occur over a relatively low number of load cycles due to low cycle fatigue [33]. In addition to recognizing the effect of overload on WTG bearing premature failures, a recent study analysing planet bearing reaction forces has suggested that the bearing under-loading is a significant factor as a cause of premature failures [31]. The results reveal that the planet bearings are under-loaded once per rotor cycle 40 ~ 70% of the time regardless of wind speed conditions; which may cause a loss in surface traction, sliding, and resulting surface damage accelerating fatigue failure [31].

WECs are physical cracks in the material subsurface surrounded by white etching areas (WEAs) [20]. Despite considerable evidence [19, 20, 22, 23, 34–38], there has been, as yet, no method devised to prove absolutely that butterfly cracks are indeed the point of damage initiation. Findings in Refs. [24, 38] suggest that WEAs are formed at butterflies by an evolving microstructural change leading to the nano-crystalline structure by material transfer and “rubbing” between surfaces of inclusions and the steel matrix. Impurities such as non-metallic inclusions may be initiation points due to local stress concentration, residual stress from heat treatment, the creation of free surfaces during quenching, and/or dislocation accumulation [34]. The work reported in Ref. [5] shows that WEC networks observed in the failed bearings from the field returned WTGs preferentially initiate as butterfly cracks, before propagating away from the initial butterfly into a broader WEC network. WEAs form adjacent to micro-cracks, or possibly form first and promote micro-crack growth. Evidence that micro-cracks precede the formation of the attached WEA is presented in Ref. [39], where RCF cracks were created in 100Cr6 bearing steel with artificially introduced voids, appearing before the WEA. This evidence suggests that it is the repeated contact of the free surfaces, formed at micro-cracks, against each other, that leads to the deformation and hardening required to create the WEA microstructure [37]. A recent study captures the evolution of WEC formation from subsurface initiation at a non-metallic inclusion and crack propagation to the contact surface, eventually causing flaking, providing evidence that the crack is a prerequisite of the microstructural alteration [40].

Significant surface sliding between bearing rollers and raceways, defined as the slide-to-roll ratio (SRR), at low load and high-speed conditions during rapid transient accelerations and decelerations has been reported by using a dynamic bearing model [41]. A number of investigations have been conducted to reproduce WECs and WEAs using test rigs under laboratory conditions of varying levels of contact pressure and surface sliding. RCF tests have been carried out on cylindrical roller thrust bearings and WSF is formed at a low contact pressure of 1200 MPa and surface sliding of up to ± 14.8%, confirming that subsurface crack initiation and propagation of WECs is at least one of the mechanisms of WSF in bearing steels [42]. Using a three ring on roller test rig, WECs have been created on bearing steel samples that experience negative sliding when the SRR is set at − 30% under contact pressure of 1.9 GPa [43]. It is stated that negative sliding acts to close a surface crack, as the roller is moving faster than the rings, leading to increased crack surface rubbing and the energy generated by this rubbing is the dominant cause of the steel recrystallization resulting in WEAs. A follow-on study of the work in Ref. [44]. found a correlation between the presence of WECs and the cumulative frictional heat energy. A four-stage WEC initiation process is proposed, consisting of the presence of dark etching areas (DEAs), cracking through DEAs, cracking with mixed WEA and DEA, and fully developed WEC networks. These studies have provided strong evidence that sliding at the surface can be a dominant driver in the formation of WECs [43].

**Fig. 1:**
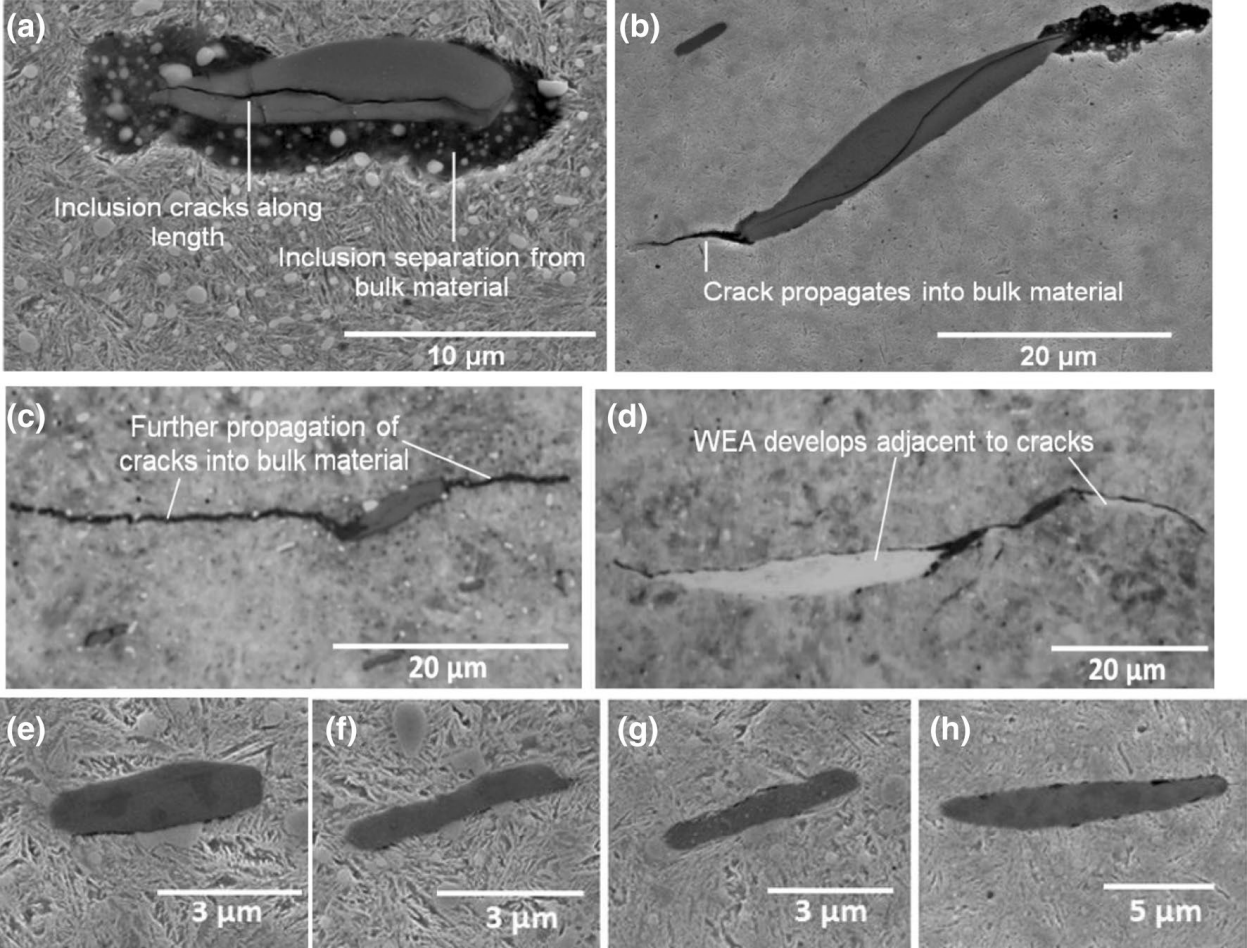
Four different forms of subsurface-damaged MnS inclusions from a failed WTG planetary bearing: **a** separation of inclusion from the surrounding steel matrix; **b** internal cracking of inclusion; **c** crack propagation into surrounding material; **d** WEAs attached to these cracks and/or free surfaces; and **e**–**h** examples of undamaged inclusions at depths of greater than 5 mm from contact surface

All types of non-metallic inclusions in bearing steel may act as crack initiation sites under high enough contact stress [34]. In 100Cr6 bearing steel, evidence is available to confirm that manganese sulphide (MnS) inclusions are the most likely to interact with butterfly cracks [20, 23, 37]. By destructively sectioning failed inner raceways from field-returned WTG planetary bearings, micro-cracks and WEAs were found attached to MnS inclusions at various subsurface depths. Four different forms of damage have been found at MnS inclusions as shown in Fig. 1 [45, 46], these are (a) separation of the inclusion boundary from the surrounding steel matrix creating free surfaces; (b) the internal cracking of the inclusion creating free surfaces; (c) crack propagation from these free surfaces into surrounding material; and (d) the development of WEAs attached to these cracks and/or free surfaces. Most recent studies on failed bearings from field returned WTGs have shown WECs preferentially initiate as butterfly cracks at dual-phase inclusions of aluminium with sulphur and manganese [47], around oxide and dual-phase inclusions [48], and at Type D globular duplex inclusions especially when an inclusion has low aspect ratio (ratio of inclusion lengths along major to minor axes) of 2:1 [49].

### Hypothesis of Inclusion-Initiated Micro-Cracks and WEAs

In this study, a hypothesis of the effect of impact loading and rolling contact fatigue on damage initiation at non-metallic inclusions is proposed. It focuses on the investigation of micro-crack initiation and WEA formation at non-metallic inclusions due to factors related to WTG bearing impact loading and surface sliding, and their interactions with non-metallic inclusions.

### The Effect of Impact Loading

It is important to consider the effect of transient operating conditions, prevailing in the WTG application, such as impact loading, on premature bearing failure. The local deformations near the contact point that arise during impact vary according to the relative velocity at the point of initial contact as well as the hardness of the colliding bodies. For WTG bearings, low-speed impact velocities (below 1 m/s) result in contact pressures that cause only small deformations in a small region adjacent to the impacted area. During a very short period of contact, stresses generated in the contact decrease rapidly with increasing radial distance, so the internal energy of deformation is concentrated in a small region immediately surrounding the contact [50]. For contact bodies that are surface hardened, only very small deformations are required to generate very large contact pressures. Hence, in a small region surrounding the contact area, the colliding bodies are subjected to large stress and corresponding strains that can exceed the yield strength of the material, possibly around subsurface material defects such as inclusions.

**Fig. 2:**
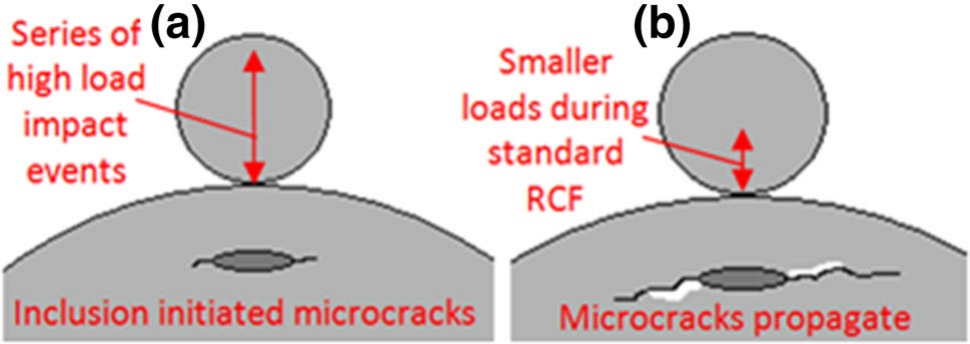
Importance of impact loading (not to scale), showing a high-impact load event that initiates subsurface micro-cracking, which is subsequently propagated by standard RCF loading

It has been hypothesized that impact loading is a key factor affecting subsurface damage initiation at inclusions [51]. Figure 2 describes a hypothesis, showing that if a high-impact load event happens prior to a series of lesser loading events, then the damage is more serious than that if these events occur in the reverse order, due to the initiation of fatigue cracking at inclusions (this will be analysed in detail in Sect. 2).

### The Effect of Surface Traction

**Fig. 3:**
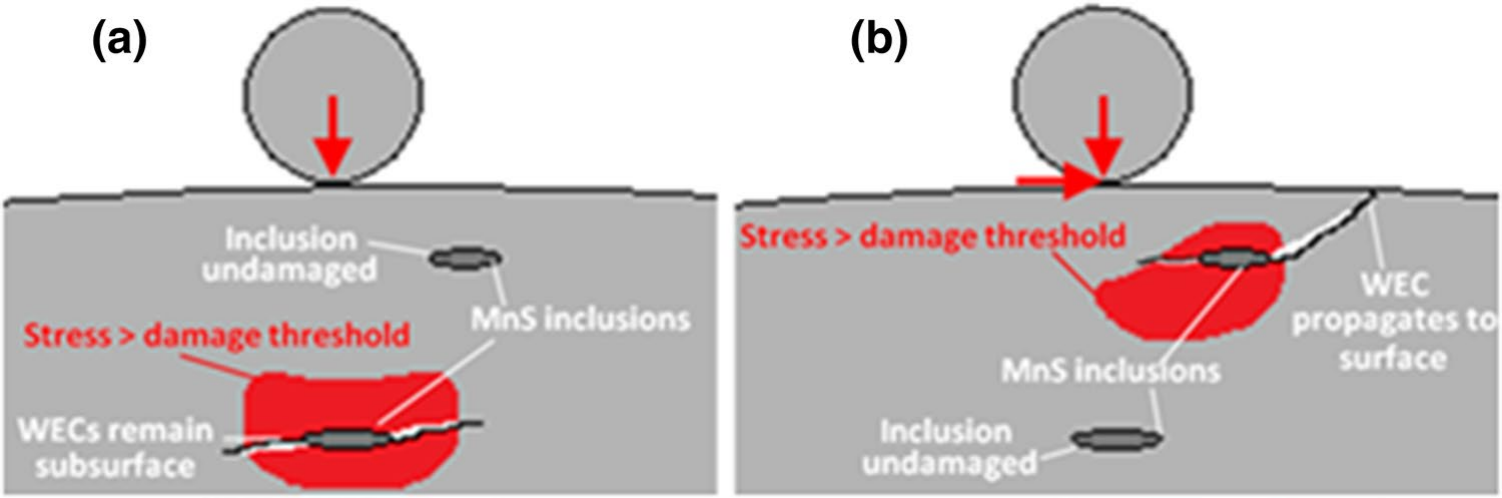
Importance of surface traction (not to scale): **a** pure rolling: critical stress threshold exceeded relatively deep—micro-cracking at inclusion may never reach surface and cause failure; **b** rolling and sliding: critical stress field shifted closer to surface—micro-cracking at inclusion propagates to surface, leading to failure

The importance of the surface traction caused by sliding between rolling elements and raceways for general applications has been documented [52, 53]. As discussed early, for WTG bearings, significant sliding due to transient operation conditions has been reported and it has been recognized that the surface sliding has an important effect on initiating micro-cracks at subsurface inclusions leading to surface spalling [31, 32, 41–44]. Since surface traction shifts the subsurface high-level stress field closer to the surface, the locations of maximum shear stresses occur at shallower depths. Since cracks have to propagate a lesser distance from shallower inclusions, those cracks initiated at shallower depths have a higher probability of leading to surface damage. The potential importance of surface traction is illustrated in Fig. 3 and will be analysed in detail in Sect. 2.

### The Effect of Subsurface Inclusions

Additional considerations arise when considering the effects of impact loading and surface traction on stress concentrations at material defects. The previous work in this area tends to ignore the effect of surface traction on the position of maximum shear stress at material defects and omission of large contact pressure on a small concentrated contact area due to impact loading. It is believed that the combined effect of high levels of contact pressure and surface traction interacting with inclusions located at a critical position in subsurface of the contact area is one of the key factors contributing to micro-crack initiation and WEA formation at inclusions leading to the premature failure of WTG bearings.

To investigate the proposed hypotheses, this study has developed experimental testing methods to recreate micro-crack initiation and WEA formation at inclusions in bearing steel specimens. A repeated impact test was developed to cause high stress levels locally at inclusions in the subsurface and to initiate damage at inclusions. Both normal impact and oblique impact tests were designed in order to investigate the effect of surface traction. Varying contact pressures and SRRs were applied in order to determine thresholds at which resulting damage at inclusions was observed. Rolling contact fatigue testing of twin-disc specimens that had been pre-damaged by impact loading, as well as specimens that had not, was carried out in order to investigate effects of impact loading and surface traction as illustrated in Figs. 2 and 3. The RCF experiment also explored damage thresholds for surface traction and contact pressure.

## Analysis of Effects of Subsurface Stresses and Inclusions on Damage Initiation

To design test samples and to determine key testing variables, subsurface stresses of contact bodies under both rolling contact and impact loading conditions have been calculated. Hertzian static contact theory is used to calculate subsurface stresses when subjected to various levels of contact pressure and surface traction. Rigid body impact theory is used to determine the impact energy required to induce sufficient high stress levels around inclusions to initiate fatigue cracks. A method based on small crack fracture mechanics is applied to estimate the fatigue limit and threshold stress for non-propagation of micro-cracks at inclusions, in order to predict if an inclusion can become a fracture origin.

### Subsurface Stresses Under Normal and Tractive Loads

Hertzian static contact theory is widely used to approximate the magnitude and location of subsurface stress in rolling contact [54]. In the case of the WTG planetary bearings considered in this study, non-conforming line contacts between the inner raceway and rolling elements were assumed. Under a combined normal and surface tractive load, expressed as a normal contact pressure distribution *p*_*N*_ and surface tractive pressure *p*_T_, for any point (*x, z*) within the subsurface of the raceway, the stress components in *x-z* coordinates can be determined by

1 $${\sigma _{xx}}= - \frac{{2z}}{\pi }\mathop \smallint \limits_{{ - a}}^{a} \frac{{{{\left( {x - s} \right)}^2}}}{{{{\left\{ {{{\left( {x - s} \right)}^2}+{z^2}} \right\}}^2}}}{p_N}\left( s \right){\text{ds}} - \frac{2}{\pi }\mathop \smallint \limits_{{ - a}}^{a} \frac{{{{\left( {x - s} \right)}^3}}}{{{{\left\{ {{{\left( {x - s} \right)}^2}+{z^2}} \right\}}^2}}}{p_{\text{T}}}\left( s \right){\text{ds}}~~$$

2 $${\sigma _{zz}}= - \frac{{2{z^3}}}{\pi }\mathop \smallint \limits_{{ - a}}^{a} \frac{1}{{{{\left\{ {{{\left( {x - s} \right)}^2}+{z^2}} \right\}}^2}}}{p_N}\left( s \right){\text{ds}} - \frac{{2{z^2}}}{\pi }\mathop \smallint \limits_{{ - a}}^{a} \frac{{(x - s)}}{{{{\left\{ {{{\left( {x - s} \right)}^2}+{z^2}} \right\}}^2}}}{p_{\text{T}}}\left( s \right){\text{ds}}$$

3 $${\tau _{xz}}= - \frac{{2{z^2}}}{\pi }\mathop \smallint \limits_{{ - a}}^{a} \frac{{\left( {x - s} \right)}}{{{{\left\{ {{{\left( {x - s} \right)}^2}+{z^2}} \right\}}^2}}}{p_N}\left( s \right){\text{ds}} - \frac{{2z}}{\pi }\mathop \smallint \limits_{{ - a}}^{a} \frac{{{{\left( {x - s} \right)}^2}}}{{{{\left\{ {{{\left( {x - s} \right)}^2}+{z^2}} \right\}}^2}}}{p_{\text{T}}}\left( s \right){\text{ds}}~$$

4 $${p_N}\left( x \right)={p_0}\sqrt {1 - \frac{{{x^2}}}{{{a^2}}}} ~,$$

where *a* is the half of contact width, and *x* is the coordinate tangential to the normal direction of contact surfaces, defined by the *z*-axis. Thus, the contact pressure rises from zero at the edges of the contact region (where *x* = ± *a, z* = *0*) to the peak contact pressure, *p*_0_, at the centre of the contact (*x* = *0, z* = *0*). The surface tractive pressure *p*_T_ is determined by the normal contact pressure *p*_*N*_ and the traction coefficient *µ*_*T*_, i.e. *p*_T_ = *µ*_T_*p*_*N*_.

**Fig. 4:**
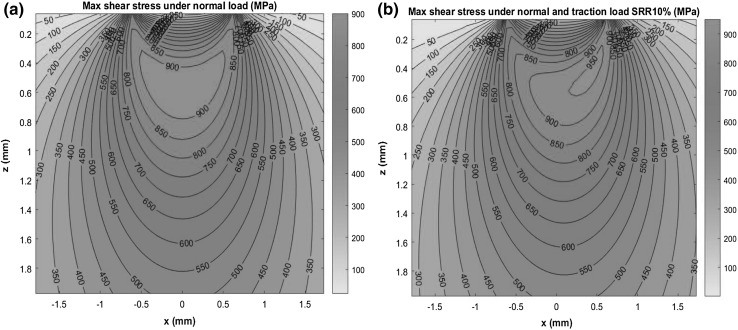
Subsurface maximum shear stress distributions: **a** under normal load; **b** under combined normal and tractive load (*p*_0_ = 3030 MPa, *a* = 0.6171 mm, SRR = 10%)

From the stress components determined from Eqs. (1), (2), and (3), the maximum shear stress, τ_max_, and the maximum orthogonal shear stress, τ_O, max_, can be determined. For contact surfaces of two identical cylindrical discs of radius 23.5 mm, Fig. 4 shows an example of the calculated subsurface maximum shear stress distributions under normal contact load only (*p*_*N*_) and under combined normal contact and surface traction loads (*p*_*N*_ and *p*_*T*_). When under a normal contact load only, the maximum shear stress zones of 900 MPa are positioned at distance from the contact surface at *z* ≈ 380 ~ 800 μm (Fig. 4a). When the same normal contact load is combined with a surface traction load using SRR = 10%, the maximum shear stress zones are shifted towards the direction of traction load, closer to the contact surface. The maximum shear stress zones of 900 ~ 950 MPa are now positioned at the distance *z* = 0 ~ 800 μm, expanding and reaching the contact surface (Fig. 4b).

### Subsurface Stresses due to Impact Loading

For the hard body contacts in a WTG rolling element bearing, it is considered that elastic-perfectly plastic material can be assumed for the analysis of local deformation under elastic impact and the initiation of plastic deformation. First developed by Hertz this quasi-static theory for elastic deformation localized near the contact area provides a good approximation for collisions of hard bodies where the contact region remains small in comparison with the size of either body [50]. For elastic impact, under an impact velocity *u*_0_, the kinetic energy, *T*_0_, of normal relative motion can be calculated to determine the largest contact force at the centre of the contact, *F*_I_, the radius of the contact region, *r*_a_, and the elliptical distribution of Hertzian contact pressure$~{p_N}\left( r \right)$ over the contact region, *r*, as detailed in Eqs. (5), (6), and (7) [50]:

5 $${F_{\text{I}}}=1.94 \times T_{0}^{{3/5}}(E_{{12}}^{{2/5}}R_{{12}}^{{1/5}})$$

6 $${r_{\text{a}}}=0.91 \times {({F_{\text{I}}}{R_{12}}/{E_{12}})^{1/3}}$$

7 $${p_N}\left( r \right)={p_0}\sqrt {1 - \frac{{{r^2}}}{{r_{{\text{a}}}^{2}}}} ,~~~~~{\text{the}}\,{\text{max}}\,{\text{pressure}}\,{\text{at}}\,{\text{the}}\,{\text{centre}}\,{\text{of}}\,{\text{contact}}~~\,{p_0}=\frac{{1.5{F_{\text{I}}}}}{{\pi r_{{\text{a}}}^{2}}}~~,$$

where *E*_12_ and *R*_12_ are the effective Young’s modulus and effective radius of contact bodies, respectively.

In order to consider the effect of surface traction during impact, oblique impact is also considered in this study. For oblique impact, both normal and frictional forces contribute to the local subsurface stress field. It is assumed that the tangential force in the contact region is due to dry friction only; and this can be represented by Coulomb’s law with a single coefficient of friction [55]. In the case considered in this study, for low-impact velocities and two contact bodies of similar size and mass, the loss of energy to elastic waves is negligible and thus the effect of friction on the normal force can be neglected. Based on these assumptions, the tangential force during oblique impact can be expressed as the surface tractive pressure *p*_T_ determined by the normal contact pressure *p*_*N*_ and coefficient of friction *f*, i.e. *p*_T_ = *f p*_*N*_. Based on *p*_*N*_ and *p*_T_ determined, the subsurface stress fields of contact surfaces due to oblique impact can be determined using Eqs. (1), (2), and (3), similar to that described in Sect. 2.1.

### Effects of Inclusions on Fatigue Limit

The effect of small defects and notches on fatigue limit has been investigated extensively; however, there is no reliable quantitative method to evaluate the effects of non-metallic inclusions [56, 57]. There are many factors that affect the fatigue limit of bearing steel, these include inclusion characteristics (shape, size, depth, and orientation to the surface), adhesion or separation of inclusions from the matrix [46, 49], microstructure hardness, and mechanical properties of the material. Murakami [57] performed extensive investigations regarding effects of small defects and non-metallic inclusions on fatigue limit of steels, ascertaining that they were essentially the small crack problem. The proposed fatigue fracture mechanism due to non-metallic inclusions was when the nominal stress around an inclusion exceeded the material fatigue limit, the stress threshold for crack propagation at the inclusion was deemed to have been reached. The inclusion became a fracture origin and a fatigue crack was nucleated, either at the interface between the inclusion and the matrix or the inclusion itself was cracked. The crack then extended into the microstructure, resulting in final fracture [57]. A quantitative relation was established [57] to estimate the fatigue limit, *σ*_w_, considering effects of the microstructure hardness, Hv, and inclusion size, which was expressed by inclusion area:

8 $${\sigma _{\text{w}}}=\frac{{1.56\left( {{H_{\text{V}}}+120} \right)}}{{{{(\sqrt {{\text{area}}} )}^{1/6}}}},~~~{\text{area}}=\frac{{\pi \times ({\text{inclusion}}\,{\text{length}} \times {\text{inclusion}}\,{\text{width}})~}}{4}~,$$

where the inclusion area is determined by the inclusion dimensions along its major and minor axes. Murakami stated that correlation between this quantitative relation and experimental fatigue test data was observed for Hv values ranged from 70 to 720 and $\sqrt {{\text{area}}}$ was less than 1000 μm, under various loading conditions of rotating bending and torsion for various materials. As discussed in Sects. 2.1 and 2.2 and shown in Fig. 4, under oblique impact and RCF loading conditions investigated in this study, the subsurface material of contact region was subjected to compressive stress and surface shearing conditions. No quantitative assessment method for the fatigue limit with consideration of non-metallic inclusions has been reported for complex stress states. Therefore, Murakami’s Eq. (8) has been used in this study to estimate the fatigue limit based on the inclusion sizes observed in the tested specimens. The maximum shear stress, *τ*_max_, was used as the nominal stress around inclusions thus enabled the prediction if an inclusion that could become a fracture origin. The ratio of the nominal stress to the fatigue limit, *τ*_max_/*σ*_w_, was then evaluated, when this ratio exceeds one, the inclusion is predicted to become a fracture origin [57] because *τ*_max_ at the inclusion has reached its stress threshold.

**Fig. 5:**
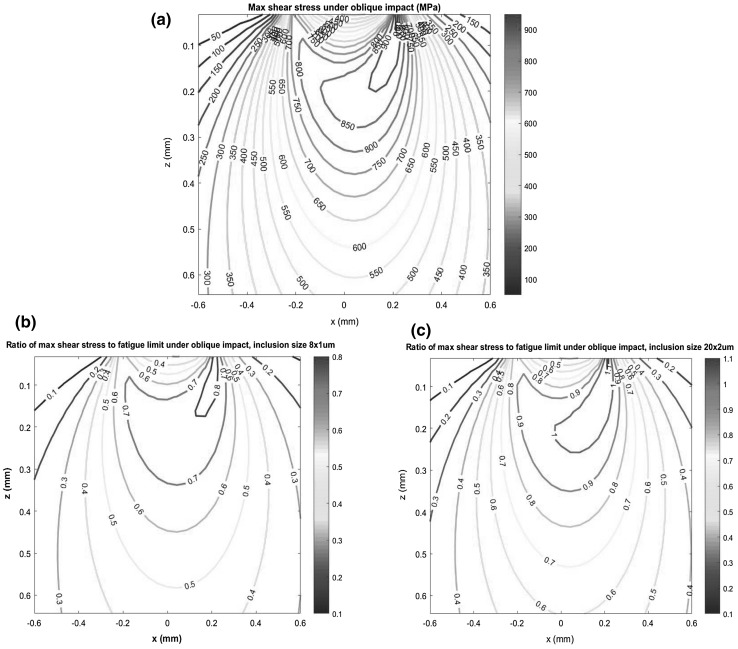
Under oblique impact: maximum shear stress *τ*_max_ and ratio of maximum shear stress to fatigue limit *τ*_max_/*σ*_w_: **a** max shear stress *τ*_max_; **b** ratio *τ*_max_/*σ*_w_ for inclusion size 8 μm × 1 μm; **c** ratio *τ*_max_/σ_*w*_ for inclusion size 20 μm × 2 μm. (*u*_0_ = 0.3535 m/s, *p*_0_ = 2759 MPa, *r*_a_=0.2145 mm, coefficient of friction = 0.1, impact angle 45°; steel hardness *H*v = 760)

Figure 5 shows distributions of the subsurface maximum shear stress and ratio of the maximum shear stress to the fatigue limit under oblique impact with normal impact velocity of 0.3535 m/s, between two identical discs of radius of 23.5 mm, made from 100Cr6 bearing steel. The impact induced the largest contact force of *F*_I_ = 265.9N and the radius of the contact region of *r*_a_ = 0.2145 mm, calculated from Eqs. (5) and (6). This resulted in a peak contact pressure *p*_0_ = 2759 MPa and a maximum shear stress zone of 918 MPa in subsurface, as shown in Fig. 5a. It is clear that the impact load caused a high stress zone within a small contact region, as described in Sect. 1.2. The fatigue limit of the material was estimated by considering steel hardness Hv760 and the inclusion size in range of 8 ~ 20 μm in length and 1 ~ 2 μm in width, as that observed in the tested specimens. Figure 5b and c shows that the highest ratios of nominal stress to fatigue limit are in the range of 0.8 ~ 1.06 at varying depths up to 200 μm for the smaller inclusion size (8 μm × 1 μm) and 430 μm for the large inclusion size (20 μm × 2 μm).

## Experimental Design and Testing

**Fig. 6:**
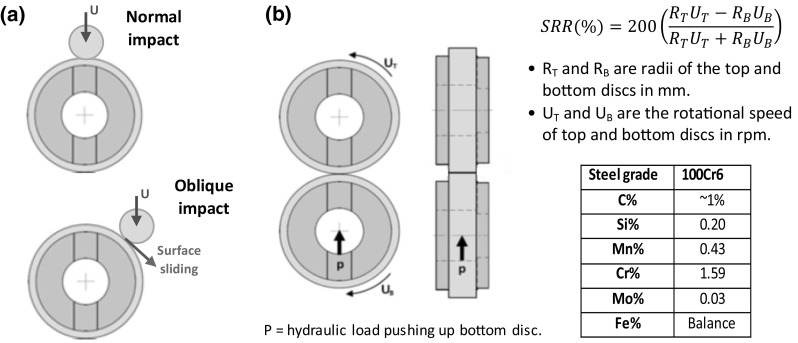
Illustration of experiments: **a** impact test; **b** rolling contact fatigue test of twin discs

Applying the methods of subsurface stress analysis and fatigue limit estimation as discussed in Sect. 2, two experimental testing methods were developed, an *Oblique Impact* test to pre-seed subsurface damage at inclusions and a rolling contact fatigue (RCF) test using twin-disc specimens. These discs were identical in radius of 23.5 mm, made from 100Cr6 bearing steel. The discs underwent a hardening heat treatment process to achieve a value of 61 HRC (or Hv760). Following heat treatment, the surface was ground to a maximum arithmetic mean surface roughness *R*_a_ of 0.39 µm and a maximum root mean squared surface roughness *R*_q_ of 0.50 µm which were determined by measurement before test. The chemical composition of the bearing steel 100Cr6 was checked using EDX analysis as shown in Fig. 6.

### Oblique Impact Tests for Pre-seeding Damage at Inclusions

The impact test rig described in Ref. [51] was used to seed damage in the test specimens at two opposite locations on the circumference of each disc representing bearing raceways. As illustrated in Fig. 6a, the impact rig can also create oblique impacts, allowing the impacting ball to hit the specimen at an angle of 45° to the tangent, introducing both normal load and surface traction. 50,000 repeated oblique impacts were applied at the impact locations. Any location further than a few millimeters away from the point of impact was assumed to be not directly affected by impact loading and so the effects of subsequent RCF tests without pre-seeded impact damage could be investigated and compared to the pre-damaged impact zone.

The maximum impact velocity in the normal direction that could be applied by the impact rig was 0.3535 m/s. This was used to pre-seed subsurface damage before RCF testing commenced, in order to initiate subsurface micro-cracks at inclusions, which were expected to propagate under RCF testing. Due to the limitation of the maximum impact velocity of the rig, the majority of the calculated values of the maximum shear stress to the fatigue limit ratio shown in Fig. 5b and c are below one. However, it is considered that the inclusions located within these zones could become facture origins due to impact loading and subsequent RCF loading cycles.

### RCF Tests for Micro-Crack Propagation at Inclusion

The RCF testing using twin discs, illustrated in Fig. 6b, was designed to investigate early stage damage after comparatively few RCF load cycles (2 × 10^5^) to that of some previous studies [22, 26, 40, 42, 43]. Considering the maximum loading capability of the twin-disc machine and contact stress levels required to initiate subsurface damage, specimens were designed to induce the maximum values of contact pressure *p*_0_, as 1.79, 2.42, and 3.03 GPa respectively. Figure 4 shows the maximum shear stress distributions when under a normal load and a combined normal and tractive load, respectively, when the contact pressure *p*_0_ is 3.03 GPa.

In the RCF test, the SRR was also changed in order to investigate the effect of changing the level of surface traction on inclusion-initiated damage. The maximum allowable SRR level of the RCF test rig was 10% thus a range of SRR from 0.2 to 10% was tested. Specimens representing raceways were positioned on the top shaft that rotated at rotational speed *U*_T_ and those representing the rollers on the bottom shaft rotated at speed *U*_B_, where *U*_T_ < *U*_B_. The lower disc can be controlled to rotate faster than the upper one in order to introduce sliding, controlled by a computer.

All other variables were held constant, including the number of pre-seeded impact cycles of 50,000. Details of the SRR and maximum contact pressure (*p*_o_) levels in each of the 12 tests are shown in Table 1. The traction coefficient (*µ*_T_) was measured during each test and mean values were found, as shown in the table. These values were used in Eqs. (1)–(3) to determine the value and location of the maximum shear stress and maximum orthogonal shear stress. After test, discs were destructively sectioned then observed and the different forms of inclusion-initiated damage were identified. Thresholds of contact pressure and SRR at which the tested discs first became damaged were identified before examining the depths at which damage occurred.

**Table 1:** RCF test parameters, traction coefficient, maximum shear stresses *τ*_max_ and *τ*_0,max_, and their depths

Disc number	1	2	3	4	5	6	7	8	9	10	11	12
SRR (%)	0.2	2	5	10	0.2	2	5	10	0.2	2	5	10
Max contact pressure, *p*_o_ (GPa)	1.79	1.79	1.79	1.79	2.42	2.42	2.42	2.42	3.03	3.03	3.03	3.03
Mean traction coefficient, *µ*_T_	0.029	0.064	0.078	0.079	0.021	0.067	0.080	0.083	0.029	0.070	0.079	0.082
Half of contact width, *a* (mm)	0.365	0.365	0.365	0.365	0.492	0.492	0.492	0.492	0.617	0.617	0.617	0.617
Max shear stress, ${\tau _{{\text{max}}}}~$ (MPa)	575	591	597	597	762	790	798	799	951	978	985	987
Depth of ${\tau _{{\text{max}}}}~$(µm)	292	292	292	292	394	394	394	394	494	494	494	494
Max orthogonal stress, ${\tau _{{\text{O,max}}}}~$ (MPa)	552	568	575	576	718	751	760	762	878	915	924	926
Depth of ${\tau _{{\text{O,max}}}}~$(µm)	146	146	146	146	197	197	197	197	247	247	247	247

## Results and Discussion

### Outline of the Observations

#### Surface Wear

Before and after testing, discs were weighed, the maximum amount of material removed was 8 mg and the minimum was 2 mg for each disc. This suggests wear levels were low and that the tests were well lubricated. After testing, the wear tracks left on the surfaces of the 12 discs (representing raceways) were examined using an optical microscope. No signs of surface spall were observed and there was no sign of the mark left by impact testing on the specimen surface, although small “stripes” around the circumference showing signs of scuffing were apparent in the specimens exposed to the highest surface traction levels (SRR = 5 and 10%) at higher contact pressure levels (*p*_o_ = 2.41 and 3.03 GPa).

#### Surface and Subsurface Cracks

**Fig. 7:**
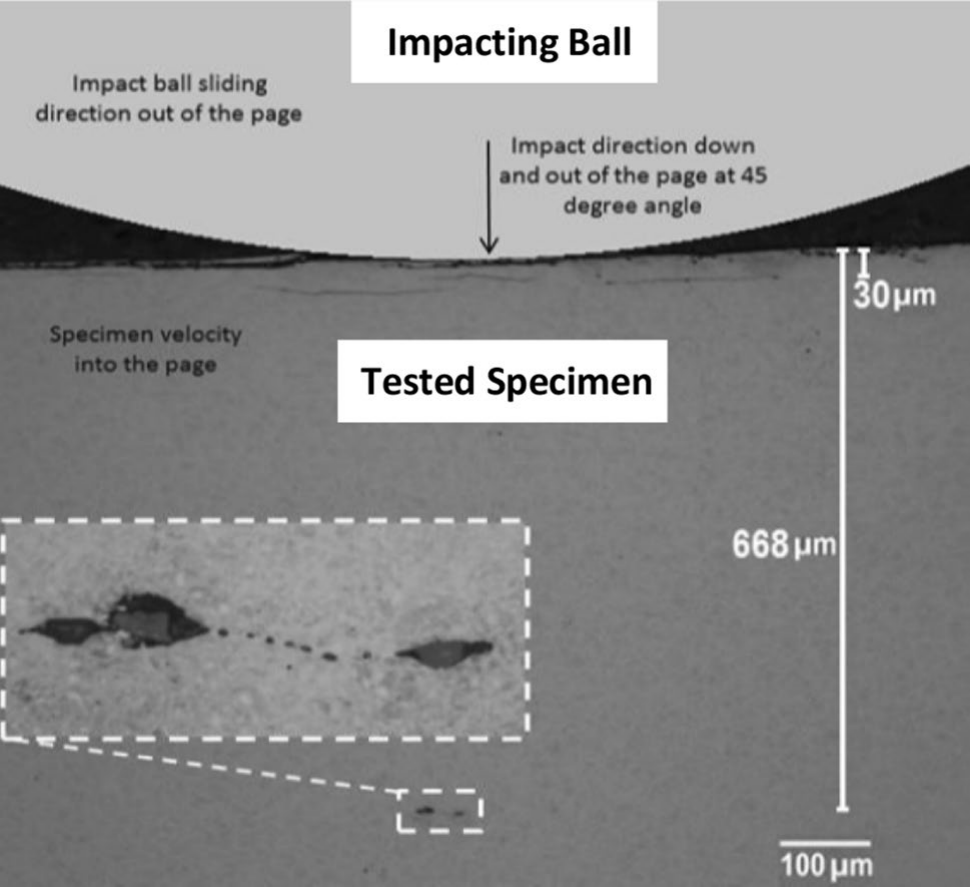
Subsurface cracks beneath impact zone of Disc 11 (with approximate position of impacting ball superimposed to illustrate the scale of the ball in comparison to the cracks)

Cracks that were linked to the surface were found in specimens that had been subjected to high surface traction and high contact pressure. Subsurface cracks, which did not appear to have any link to the surface, were found in many of the specimens. Figure 7 shows three subsurface cracks, a few hundred micrometres in length, directly under the impact zone found in Disc 11. Since the cracks were very near to the surface (30 μm), it seemed likely that surface traction played a role in their initiation or propagation. These cracks were much longer than any found away from the point of impact, the longest of which were of the order of tens of micrometres in length. The figure also displays a damage initiating pair of inclusions at a depth of 668 µm (greater than the depth of the maximum shear stress zones due to impact and RCF). These were the deepest damage-initiating inclusions found in the tested disc specimens.

#### Damage Initiation at Manganese Sulphide (MnS) and Other Types of Inclusions

**Fig. 8:**
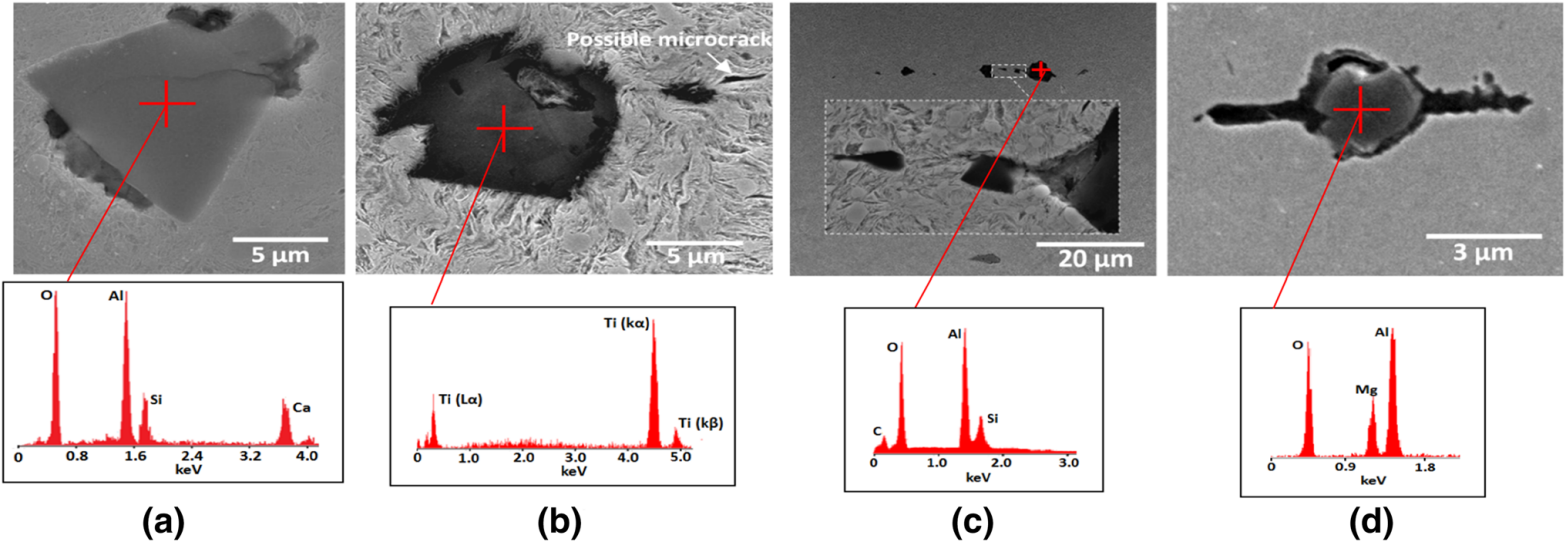
Damage at other types of inclusions and EDX analysis: **a** internal cracking of alumina inclusion; **b** micro-crack close to titanium inclusion; **c** possible damage at alumina inclusion; **d** micro-cracks at either side of alumina inclusion

**Fig. 9:**
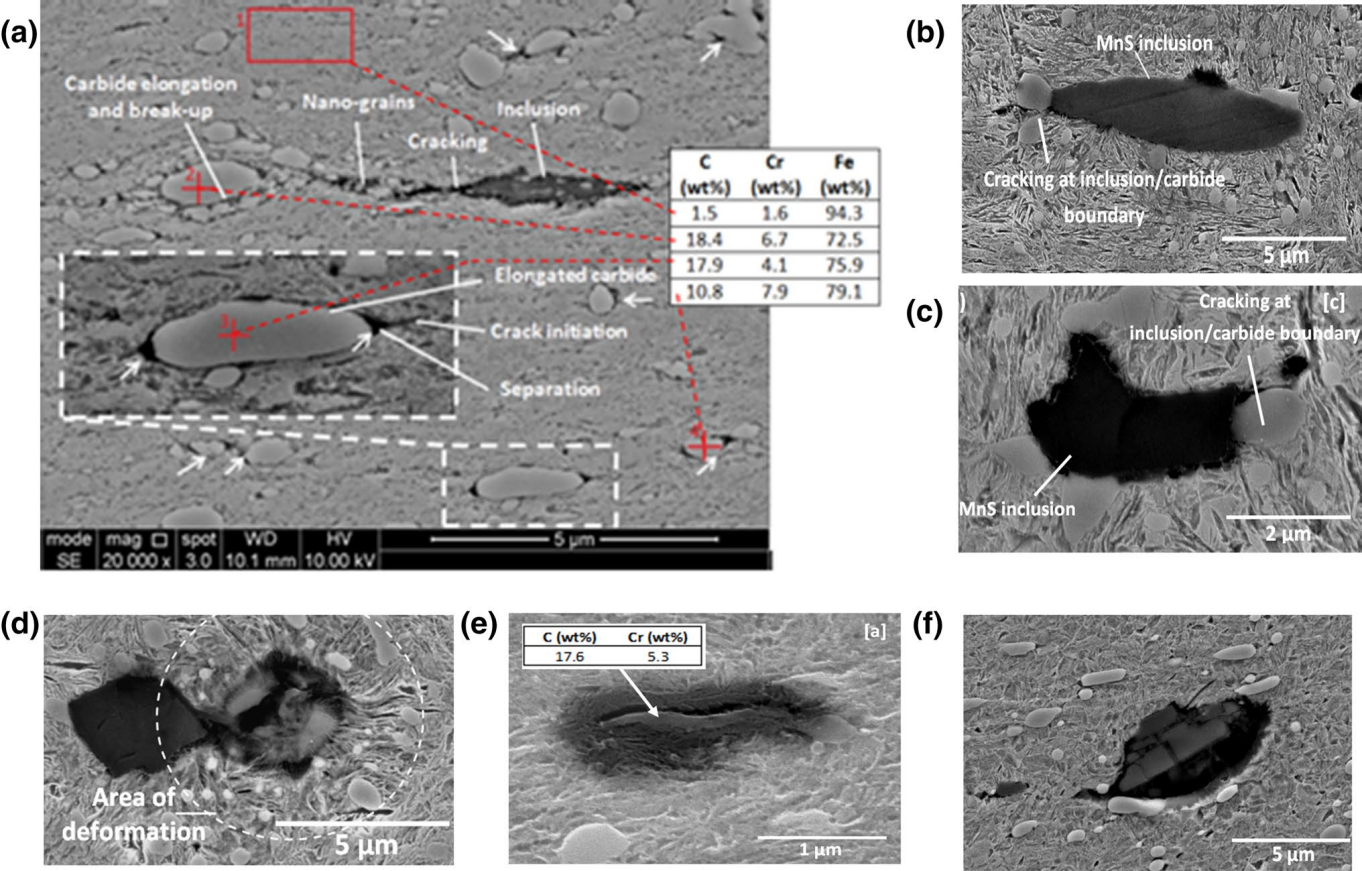
Damage at carbides and boundaries with inclusions: **a** EDX analysis of the labelled carbides (Disc 8); **b, c** cracking at MnS inclusion/carbide boundary (Discs 8 and 11); **d** dissolution of carbides in close vicinity of MnS inclusion (Disc 11); **e** severely elongated separated carbide (under impact zone of Disc 11); **f** elongated carbides around separated/cracked MnS inclusion (Disc 12)

A significant number of sites where damage had been initiated were found within the specimens of the tested discs. MnS inclusions were found to be the primary damage initiator although other forms of damage were also found including; internal cracking and external cracking at other inclusion types, carbide elongation, and possible crack initiation at carbides. Figure 8 shows some examples of damage initiation at alumina and titanium inclusions with their EDX analysis while Fig. 9 shows damage at carbides. In general, the main forms of damage were observed at MnS inclusions, which were similar to those found in both failed WTG bearings [45, 46, 49] as shown in Fig. 1 and tested specimens [51].

#### Impact Loading

Damage was found both underneath locations that had been pre-damaged by impact loading and at locations where they had experienced only RCF and surface traction. No major differences were found when observing circumferentially sectioned specimens compared to those that were axially sectioned. This may be because the damage found was at an earlier stage than that found in the failed WTG bearing and/or because the inclusion geometry varied less in the tested specimens than that in the WTG bearing. All forms of damage were more likely to occur in specimens that had been exposed to higher contact pressure and surface traction. Pre-seeding the specimens with impact loading, in general, accelerated the inclusion-initiated damage, allowing separation, internal cracking, and crack propagation to occur at lower loads and sliding levels. WEA damage was recreated but was believed to be at a much earlier stage than that in the WTG bearing.

In the following sections, observations of the subsurface inclusion-initiated damage in the tested specimens will be discussed in detail and compared with that observed in the failed bearing shown in Fig. 1. Four different forms of inclusion-initiated damage were observed in the specimens of the tested discs. These were named as Damage I: Separation at inclusion/steel matrix boundary; Damage II: Crack initiation from inclusion tips; Damage III: Internal cracking of inclusions; and Damage IV: WEA formation at inclusions. These are shown in Tables 2 and 3 and will be discussed in Sects. 4.2 and 4.3.

**Table 2:**
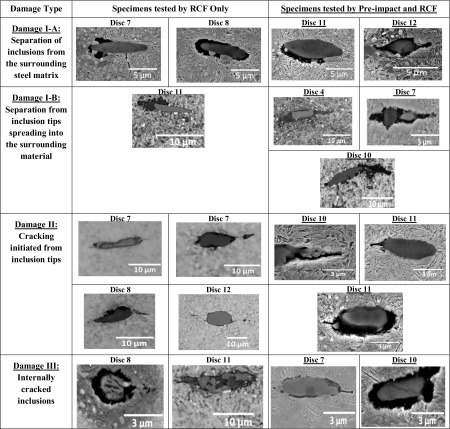
Inclusion-initiated damage in specimens tested by RCF only and Pre-impact and RCF

**Table 3:**
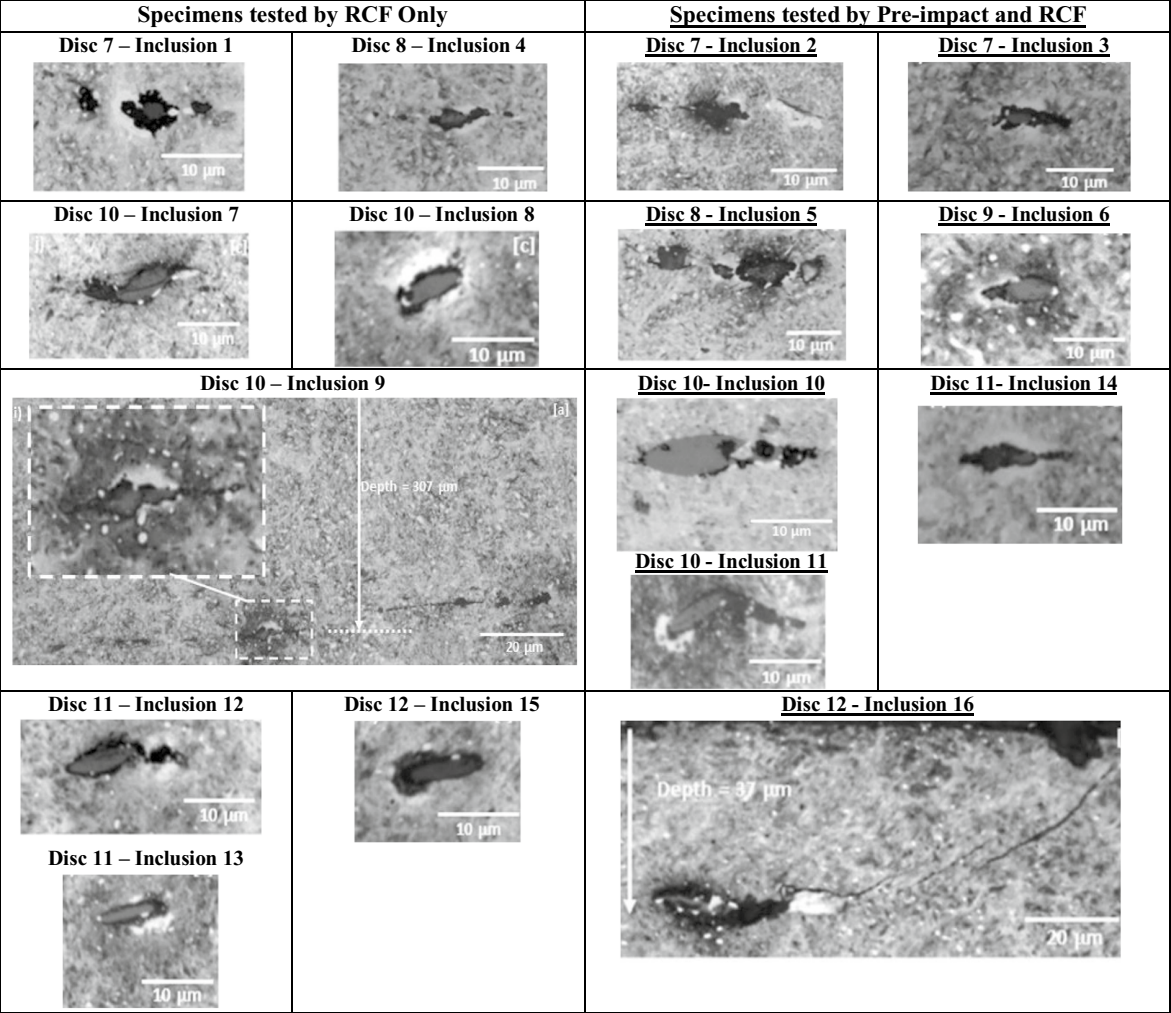
Sixteen WEAs found initiated at inclusions—Damage IV

### Separation and Cracking at Inclusions

The creation of a free surface by separation of inclusions from the surrounding steel matrix (shown in Fig. 1a) has been identified as an initiation mechanism for micro-cracks and WEAs in our previous studies [45, 46, 49]. The testing of twin discs found that separation occurred to varying degrees at a wide range of surface traction and contact pressure levels. Separated inclusions were common at depths, under the impact zone of the specimens, near to the location of ${\tau _{{\text{max}}}}$. Separation was found at inclusion tips, above and below inclusions as well as completely surrounding inclusions, examples of which are shown in Table 2, Damage I-A. All specimens were carefully washed after etching to prevent etchant getting stuck in the small void to cause acid damage to the specimen thus not exaggerating separation damage.

Separation from the tips of inclusions that spread into the surrounding material, Damage I-B, was found in specimens that had experienced higher contact pressure and surface traction, as shown in Table 2. The wide separation that appears to spread from the inclusion tips into the steel matrix. This may or may not be the same feature as the thinner cracks that have initiated from the inclusion tips, Damage Type II, as shown in Table 2. This is believed to show the first stages of cracking damage at MnS inclusions [45]. This backs up previous evidence that suggests cracking precedes the formation of WEAs at MnS inclusions. Many of the damaged inclusions were internally cracked, Damage Type III, as shown in Table 2, similar to the damage at inclusions of the failed planetary WTG bearing shown in Fig. 1. No clear differences between the impact- and non-impact-damaged specimens exist. But in general, the damage appears to be more extensive in impact-damaged zones of the specimens.

### Micro-Cracks and WEAs at Inclusions

WEAs were found adjacent to many inclusions in the higher sliding and higher contact pressure tested specimens. Many inclusions had initiated WEAs in their near vicinity, Damage Type IV, as shown in Table 3. The inclusion-initiated WEAs were given Inclusion IDs, numbered according to their disc numbers, starting from the locations without pre-impact damage then to the locations pre-impact damaged. Inclusion 16, which was closer to the surface and under the point of impact in Disc 12, appears to be separated from the matrix to the right of the inclusion, and linked to a surface breaking crack, a WEA has developed adjacent to the crack. WEAs are attached to tips of internally cracked Inclusions 7, 11, and 12, which are similar in appearance to many found in the failed WTG bearings [45, 46, 49]. In addition to the WEAs attached to inclusions with internal cracks, damage linked to the WEAs including separation and crack propagation into the steel matrix were found as displayed in Table 3. However, most inclusions appear to be significantly less damaged than those observed in the failed WTG bearings and it is thought that the damage found in the tested discs are at an earlier stage of failure due to comparatively low RCF cycles of 2 × 10^5^ tested in the experiment. Again, the inclusions that are underneath the point of impact appear, in general, to show more extensive damage, particularly in discs 10, 11, and 12 which were tested under high sliding and contact pressure levels. The damage observed further proves that inclusions are points at which white etching areas may initiate.

**Fig. 10:**
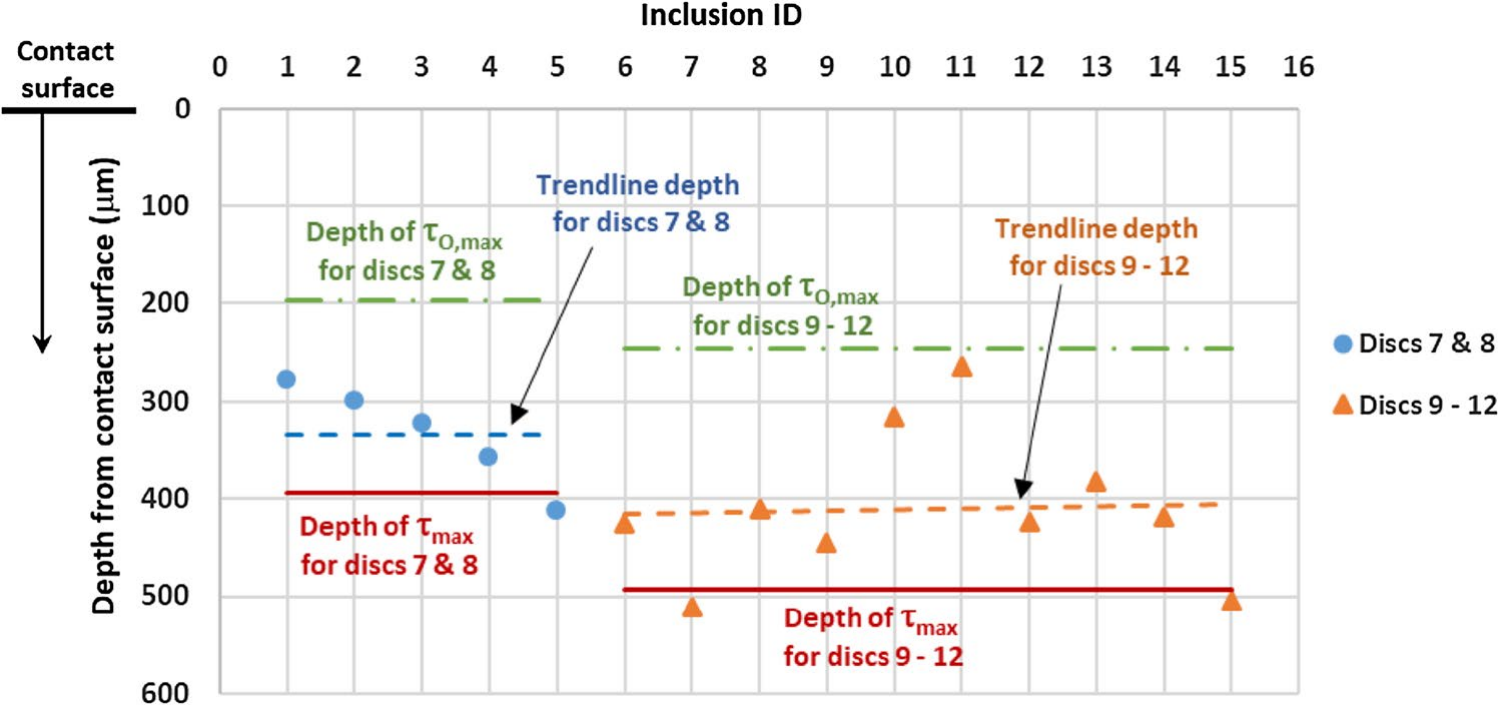
Depths of WEA-initiating inclusions in tested discs and comparison with depths of maximum shear stresses

The depth of each of the WEA-initiating inclusions was investigated. Five were found in Discs 7 and 8, which were exposed to the second highest contact pressure (2.42 GPa), and 11 in Discs 9–12, which experienced the highest contact pressure (3.03 GPa). The RCF test parameters and the calculated maximum shear stresses are shown in Table 1. The depths of each of the WEA-initiating inclusions are presented in Fig. 10, together with the depths of the RCF maximum shear stress zones, ${\tau _{{\text{max~}}}}$ and ${\tau _{{\text{0,max}}}}$. One WEA-initiating inclusion was close to the surface (Table 3, Disc 12—Inclusion 16, depth 37 μm), and was linked to a surface breaking crack. Since it cannot be proved whether the crack was initiated in the subsurface or on the surface, it was decided to ignore it when calculating the trend line depth of WEA inclusions in Discs 9–12. The trend line depths calculated from the 15 WEA-initiating inclusions are plotted in Fig. 10. For both datasets; Discs 7–8 and Discs 9–12, the trend line depth corresponded most closely with the RCF maximum shear stress, ${\tau _{{\text{max}}~}}$, however, closer to the contact surface, affected by the surface traction and impact stresses as discussed in Sect. 2 and illustrated in Figs. 4 and 5, respectively. One limitation of these results is that the test sample sizes are fairly small, since only 16 WEA-initiating inclusions were found.

### Thresholds for Inclusion-Initiated Damage

**Fig. 11:**
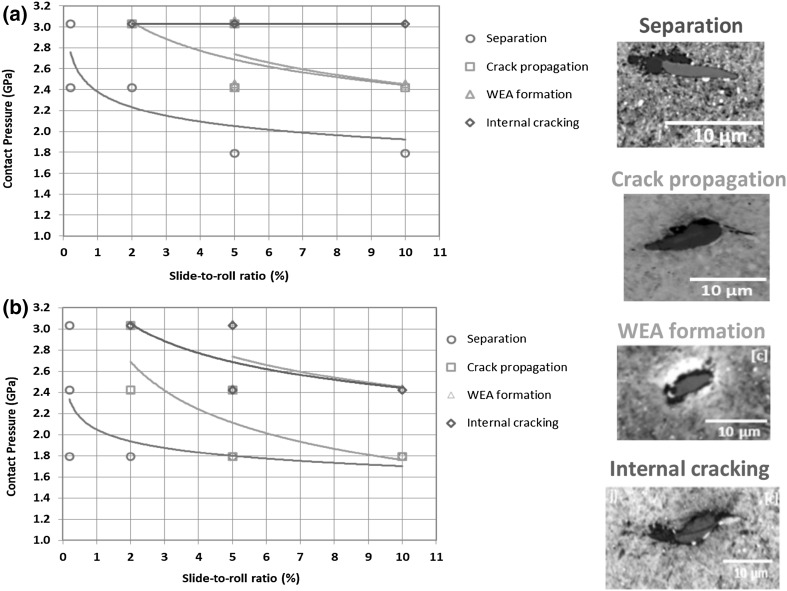
Thresholds of contact pressure and SRR for four different forms of inclusion-initiated damage: **a** specimens with no pre-seeded damage; **b** specimens pre-damaged with impact loading

**Fig. 12:**
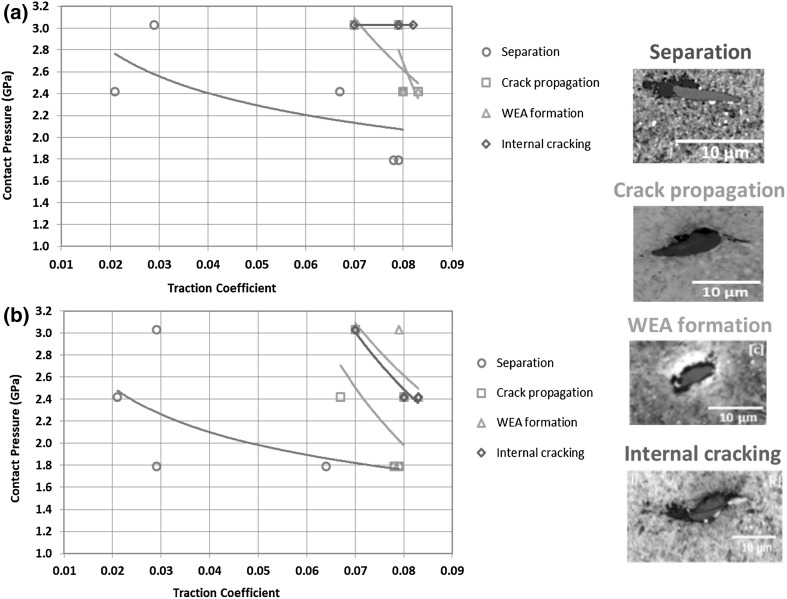
Thresholds of contact pressure and traction coefficient for four different forms of inclusion-initiated damage: **a** specimens with no pre-seeded damage, **b** specimens pre-damaged with impact loading

Thresholds of contact pressure and SRR have been identified at which the different forms of inclusion-initiated damage were found in specimens both with and without pre-seeded impact damage. For the four different forms of inclusion-initiated damage identified, Figs. 11 and 12 display the thresholds by showing the lowest contact pressures and surface tractions at which the damage was found. Figure 11 presents the thresholds of contact pressures and SRRs while Fig. 12 uses the mean traction coefficients measured during RCF testing. Comparing results in this way allows the minimum required levels of different damage-initiating factors, taking into account their combined effects, to be estimated. Any point above the plotted trend lines using the obtained data points from the tested specimens is above the threshold required to initiate damage at inclusions. It is clear that increasing contact pressure and sliding within the ranges investigated, caused increasing levels of damage. It is assumed that if a specimen has experienced damage at a lower contact pressure and sliding level, then it will do so at higher levels, even if that damage was not directly observed from the test results of this study. Clearly, these figures should be used with caution as it cannot accurately predict the thresholds between data points and contact pressures below 1.79 GPa, which were not investigated in this study. Both sets of figures show clearly that all types of damage at inclusions are sensitive to increased levels of contact pressure, SRR or traction coefficient as well as to pre-seeded impact damage. Thresholds for each form of inclusion-initiated damage will be discussed in detail in the following sections.

#### Thresholds for Initiating Inclusion Separation at Inclusion/Matrix Boundary

All damage types as discussed in Sects. 4.2 and 4.3 were more extensive under higher contact pressures and higher sliding levels. Separation at inclusion/matrix boundary (images shown in Table 2) occurred in all specimens, other than the non-impact-damaged specimens in Disc 1 (RCF test details are given in Table 1), which experienced the lowest contact pressure and lowest SRR of all RCF tests. However, increased levels of SRR above certain contact pressure levels were required for the separation to spread away from inclusion tips into the steel matrix (Damage I-B). This occurred in Disc 4 at the lowest contact pressure of 1.79 GPa (damage image is shown in Table 2, Disc 4), when SRR was 10%, as shown in Fig. 11.

#### Thresholds for Initiating Inclusion Internal Cracking

Internal cracked inclusions were not found at lower levels of contact pressure and SRR therefore it is clear that the pre-seeded impact damage was not extensive enough to directly lead to this form of damage. However, since internally cracked inclusions, images shown in Table 2, appeared at lower contact pressures of 2.42 GPa in the pre-damaged locations of discs (Fig. 11b), rather than 3.03 GPa in the locations of no pre-seeded damage (Fig. 11a), it indicates that pre-impact damage did perhaps weaken the inclusions within the impact zone. It is clear that this damage mode is affected by surface traction, since in both specimens that were pre-damaged and those that were not, a SRR threshold of greater than 2 ~ 5% appeared to exist, below which, no internally cracked inclusions were found. As shown in Figs. 11a, b and 12a, b, the thresholds of contact pressure for specimens with pre-impact damage were lowered in comparison with the trend lines for those without impact damage, indicating that the inclusion internal cracking could occur at much lower contact pressures when under higher SRRs, if the specimens were pre-damaged with impact loading.

#### Thresholds for Crack Propagation from Inclusion Tips

This form of inclusion-initiated damage appeared at higher contact pressures, and at the low contact pressure of 2.42 GPa when SRR was increased to 10%. Micro-cracks at inclusions were found to be slightly longer under the impact zone, although this result was not conclusive and may have been due to statistics of inclusion distributions as well as over-analysis of impact zone sites compared to non-impact zone sites. Comparing the thresholds of contact pressure for specimens with and without impact damage shown in Figs. 11 and 12, it was clear that the impact loading caused the threshold levels of contact pressure to be lowered considerably for initiating crack propagation from inclusion tips, specifically with the increasing levels of SRR.

As shown in Figs. 11 and 12, crack propagation from inclusion tips occurred at lower contact pressures and SRRs than that for inclusion internal cracking, which offers further evidence that the latter is not required to initiate the former [45]. It was clear that the pre-damaged specimens had lower contact pressure and surface traction thresholds for this damage mechanism to occur, further backing up the hypothesis that impact loading may accelerate damage of WTG bearings.

#### Thresholds for WEA Formation at Inclusions

As with the other forms of damage, WEA formation at inclusions only occurred above certain contact pressures and SRRs. WEA formation appeared to be less affected by the impact pre-damage. However, since only 16 WEAs were found at inclusions this result cannot be considered conclusive. It is possible that if further RCF load cycles had been applied to the specimens, the separated/crack-initiating inclusions discussed above may have gone on to initiating butterfly cracks leading to the development of WEC networks at the created free surfaces [34, 45, 46, 49]. In which case, it is likely that WEAs may have occurred at lower sliding/contact pressure thresholds if the number of RCF load cycles was higher. Evidence to support this hypothesis is presented in a study investigating the effect of impact loading on inclusion-initiated damage [51], since WEAs at inclusions were only found in the long-term impact tests with high load cycles.

## Conclusions

Threshold maps for subsurface inclusion-initiated micro-cracks and WEAs in bearing steel specimens have been investigated by RCF tests at a range of surface sliding levels and contact pressures, some of which had been pre-damaged with 50,000 oblique impact loading cycles. It has been shown that all forms of inclusion-initiated damage increased with increased levels of surface traction, contact pressure, and impact loading. The following conclusions can be reached:

- WEAs were formed adjacent to inclusions in specimens that had experienced contact pressures above 2.42 GPa and a SRR above 5%. WEA initiation was more likely to occur at higher levels of contact pressures and surface traction.
- Three other forms of inclusion-initiated damage were also observed namely; separation at the inclusion/steel matrix boundary, propagation of cracks from the inclusion tips, and internal cracking of the inclusion. All three damage forms were more likely to occur at higher contact pressures and SRRs and were all accelerated by impact loading. Impact loading had shown to lower the thresholds of contact pressure considerably for all three forms of inclusion-initiated damage, when subjected to higher SRRs.
- Surface traction accelerated inclusion-initiated WEAs and possibly it was required for them to form, since inclusion-initiated WEAs were only produced in specimens of the discs tested under RCF with high SRRs, with or without pre-damaged by the oblique impact loading. No inclusion-initiated WEAs were found in specimens that had not experienced high surface traction.
- The observation of the WEA formation at inclusions only occurring above certain SRRs implied that high levels of surface traction between free surfaces was required to create WEAs, possibly due to subsurface rubbing of two free surfaces, either at a crack or at an area of separation between damaged inclusions and the steel matrix.
- Inclusions do not need to be internally cracked to initiate micro-cracks at inclusion tips, which is different to the findings of some of the previous investigations, although the findings in this study agree that the internal cracking of inclusions certainly does lead to the initiation of some fully developed WEC networks.
